# Studying the effect of abdominal massage on the gastric residual volume in patients hospitalized in intensive care units

**DOI:** 10.1186/s40560-018-0317-5

**Published:** 2018-08-10

**Authors:** Farzad Momenfar, Alireza Abdi, Nader Salari, Ali Soroush, Behzad Hemmatpour

**Affiliations:** 1 Nursing and Midwifery School, Students Research Committee, Kermanshah University of Medical Sciences, Kermanshah, Iran; 2 Department of Sports Medicine and Rehabilitation, Imam Reza Hospital, Kermanshah University of Medical Sciences (KUMS), Kermanshah, Iran; 3 Department of Anesthesiology, Taleghani Hospital, Kermanshah University of Medical Sciences, Kermanshah, Iran; 4 Nursing and Midwifery school, Kermanshah university of medical sciences, Kermanshah, Iran

**Keywords:** Abdominal massage, Residual volume, Intensive care unit

## Abstract

**Background:**

The main problem of hospitalized patients in intensive care units is feeding, and if the patient does not receive the daily caloric intake required to his body, he will have malnutrition and problems related to it. Abdominal massage is a method used to improve digestive function in various studies, but few studies have been conducted in intensive care units, and sometimes, contradictory results have been obtained. Therefore, the present study is conducted with the aim of determining the effect of abdominal massage on the gastric residual volume in patients hospitalized in intensive care units.

**Methods:**

This study was conducted as a clinical trial in Ahwaz, in 2017. Samples were 60 patients hospitalized in intensive care units who were randomly divided into case and control groups. The intervention period for the case group was 3 days and twice daily for 20 min. Measuring the gastric residual volume was investigated before the intervention and 1 hour after the second massage each day. Data were entered into the checklist designed by the researcher and were analyzed using SPSS version 24 and descriptive and inferential tests.

**Results:**

The gastric residual volume on the second and third day after the intervention was less than before the intervention (*p* value< 0.05), the gastric residual volume before intervention with after intervention in the control group during different days, on each of the 3 days after the intervention, was more than before the intervention (*p* value< 0.05), and the gastric residual volume after the intervention in different days and the mean of different days in the case group was lower than the control group (*p* value> 0.05).

**Conclusion:**

Results represent the effect of abdominal massage on reducing the gastric residual volume in patients hospitalized in intensive care units. Therefore, it is suggested that this method can be considered as a caring method in the daily care program for these patients.

**Trial registration:**

IRCT2017062134641N2, registered 26 July 2017.

## Background

Food support has a vital role in taking care of patients in intensive care units [1]. This is one of the important goals in taking care of these patients [2]. Feeding with nasogastric (NG) tube is used for patients who are unable to feed through mouth [3], and in this case, after the inserting the NG tube, during the first 24 h, the gastric residual volume (GRV) is measured every 6 h. If the GRV is greater than 250 cc, the nurse should inform the doctor for further investigation and will not receive a meal in that session [4]; even this method is omitted based on the newest guidelines, [5] but it still is implemented in Iran as a routine measure [6]. Feeding method through NG tube tract helps to maintain peristalsis, improves blood supply, and strengthens the immune system [7], and timely and adequate nutritional support plays an important role in improving recovery, reducing physiological stress, and increasing the immunity capacity [8]. Likewise, this approach accelerates wound healing, reduces the number of hospitalization days, reduces the infection risk, and in cases where the patient is hospitalized due to ulcers or injuries, reduces catabolic responses [9]. In fact, the aim of nutritional support through the NG tube for patients in intensive care units is to reduce or eliminate malnutrition because malnutrition can cause muscle atrophy and loss of body mass [7].

Among the factors that prevent adequate feeding of patients, in this way, we can refer to delay in initializing feeding, reduction in rate of gavage, not gavaging the volume prescribed by the physician, and increasing the frequency of discontinuation of feeding [10]. Nurses working in intensive care units play a key role in implementing nutritional support in patients with decreased levels of consciousness that include: timely initializing the feeding, correct feeding, surveying gastric intolerance, examining the emplacement of NG tube, determining the amount of calories necessary for the patient, and measuring the GRV [1].

Among the most important digestive complications in patients fed by the NG tube method, food intolerance and delayed gastric emptying can be referred [11]. To find out the delay in gastric emptying, usually the best way is to measure GRV bedside the patient [12]. Studies show that 10 to 63% of patients fed with this method have stomach intolerance, which causes only 43–64% of these patients receive their daily needed calories [9]. Food intolerance in these patients is associated with the increased risk of mortality and malnutrition-related complications [13], and lack of needed nutrition intake results in loss of body mass, excessive weight loss [7], progression of infection, bedsore, the increased duration of hospitalization, the increased risk of mortality, and increased costs [14], and ultimately lead to cachexia and sarcopenia in these patients [15].

Various methods have been suggested for preventing and treating food intolerance and increasing the rate of gastric emptying, among which the use of prokinetic drugs such as metoclopramide can be referred. But these drugs have many side effects such as abdominal cramps, allergies, bronchospasm, heart disorders, and disorders in pancreas [9]. For 38.8% of hospitalized patients in intensive care units in Germany, abdominal massage is used for managing complications following immobility and improving food tolerance [9]. In this regard, the research conducted by Kahraman and Ozdemir [16] showed that the GRV on the last day had a significant reduction compared to the first day. But in the study of Tekgündüz et al. [17], the GRV after abdominal massage did not show any difference between the two test and control groups. Other researchers also believed that abdominal massage with stimulated peristaltic movements of digestive system, altered intra-abdominal pressure and induced mechanical and reflexive effects on the intestines, reducing the transit time of nutrition in the intestines and increasing the number of intestinal movements and easier food movement along the gastrointestinal tract can be considered as a palliative treatment to prevent the complications caused by this feeding method [9, 18]. Considering the few studies in intensive care units and existing controversy studies, the present study was conducted with the aim of determining the effect of abdominal massage on the GRV in patients hospitalized in intensive care units.

## Methods

This study was conducted as a clinical trial in intensive care units of Fatemeh Zahra Hospital (Ahvaz, Iran) in 2017. The research population was all the patients hospitalized in intensive care units, and samples were 60 patients hospitalized in intensive care units that were first included as convenience and then were divided into two groups by simple random method, case group (abdominal massage recipients) and control group (normal care recipients). In this method, 60 cards having the same appearance were provided, and on 30 of them, the letter A was written that identified the case group “abdominal massage,” and on the other 30, B was written to indicate the control group “usual care.” Then, another person accidentally took one of these cards, with the code written on it, so that the random allocation of patients to each group was determined.

Sample size was estimated based on the formula of comparing a quantitative feature in two groups, the 95% confidence coefficient (1-α), power 90% (1-β), as well as the mean and standard deviation (SD) of GRV in abdominal massage (105 ± 15.30 cc) and control (142.91 ± 66.7 cc) groups after intervention in Uysal et al. [9]; thus, 28 individuals were calculated to each group, so, considering the probability of attrition, 30 individuals were recruited in each group, following formula.

Inclusion criteria included having NG tube (for check the GRV), Glasgow coma scale less than 7 (because usually these patients need gavage), not having abdominal radiotherapy during last 6 weeks, and not having abdominal surgery (because the massages are forbidden for these patients). The patients who took prokinetic medications (due to interfering with massage effects) or discharged during the study (these patients did not complete the intervention course) were excluded.

To collect the data, the researcher, after obtaining permission from the Deputy of Research and Information Technology of Kermanshah University of Medical Sciences and the hospital of research location, went to the place where samples were hospitalized (intensive care units) and selected those who had the criteria for inclusion in the study, then the individual accompanied with the patient (one of the first-class relatives who was responsible for the patient) was asked to complete a written informed consent after a complete explanation of the study goals and the method of work. Samples were randomly divided into two case and control groups. Every day, the researcher went to the intensive care unit of Fatemeh Zahra Hospital in the morning from 8 am to 12 noon for a period of 3 months and conducted the study. The initial information was collected in a designed checklist, using file information and questioning from the individual accompanying the patient.

### Data collection tools

Data were collected using a researcher-designed checklist containing two parts, one including demographic information (age, sex, marital status, education, occupation) and the other for pursuing the record of gastric nutrition status (in relation to the type of nutrition, the amount of food each time, number of feeding times, number of vomiting times, GRV in different times, and medication). The form of pursuing the patient’s nutritional status was adjusted and used after a detailed study of the books and scientific publications and using available articles on the subject of the study. The qualitative content validity method was used to investigate the validity of the checklist; thus, the forms were distributed to ten faculty members of nursing and intensive care field, and their opinions were applied.

### Intervention

**Fig. 1:**
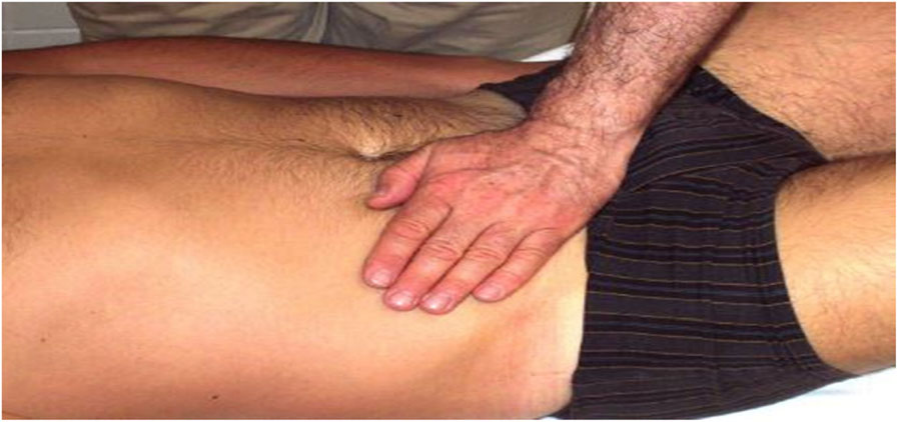
Stage one, brushing on the abdomen skin

**Fig. 2:**
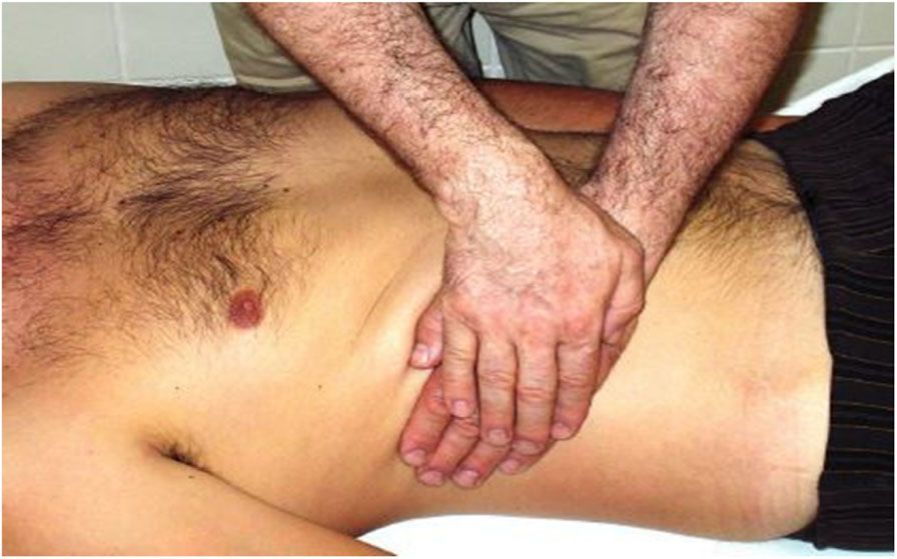
Stage two, the dominant hand is placed on the abdominal skin and the other hand on it with appropriate presser, the skin drown

**Fig. 3:**
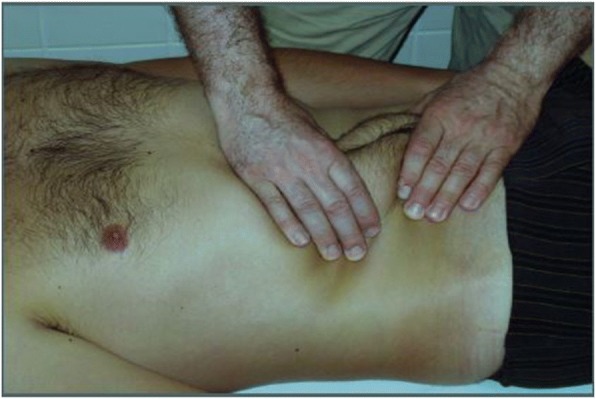
Stage three, the abdominal skin shape is changed with rubbing

**Fig. 4:**
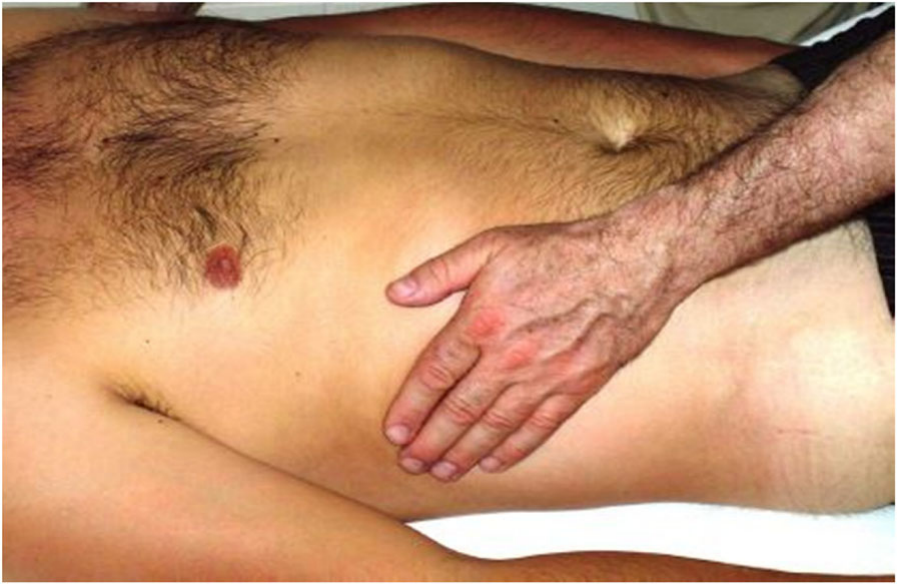
Stage four, the shake movements in line with armpit from top to down and vice versa

**Fig. 5:**
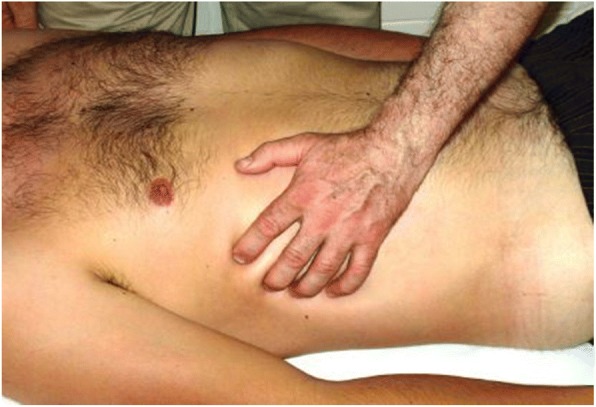
Stage five, the fingers are placed between intercostal spaces and pulled appropriately

In the present study, massages based on the tensegrity principles were used, and the primary outcome was the change of GRV after abdominal massage. The intervention period for the case group was 3 days. These patients received 20 min of abdominal massage intervention twice a day, and the interval between two massages was 2 h. Each day, before the intervention and 1 hour after the second massage, the GRV was measured and investigated. This type of massage technique consists of five steps. The first stage of massage starts with movements like brushing the skin in the abdominal area (Fig. 1); in the second stage, elastic deformation of the thoracolumbar fascia will be performed in the form of displacement, the dominant hand is placed on the abdominal skin, and the other hand is placed on it, and with an adequate pressure of hand, the skin of under pressure area is squeezed (Fig. 2). In the third stage, the skin of the abdominal skin is elastically deformed by massage, the abdominal skin is picked, and kneaded by the fingers (like kneading dough) (Fig. 3). The fourth stage involves shock movements along the armpit from top to bottom and bottom to the top (Fig. 4), and the last stage contains deformation of the muscles in the intercostal spaces of false ribs (the fingers are placed between the intercostal spaces and pulled on the skin with an appropriate pressure) (Fig. 5); the lubricant gel is used to facilitate the massaging. The patient’s position is asleep to the back while undergoing massage. The angle between the bed and the patient’s head is 30 to 45 degrees, and the patient’s legs are placed on a pillow. This condition helps to relax the abdominal muscles.

Patients hospitalized in intensive care unit were gavaged every 3 h according to the protocol, and the studied patients were fed in the same way. First, using a special 50 ml gavage syringe, 5 cc of air was quickly injected into the stomach, and using a stethoscope, the voice in the stomach was heard, and after confirmation of the insertion of the NG tube, first lavage was performed and the gastric volume residual was measured, and this amount was returned to the stomach with any amount it has, and a certain amount of food was gavaged into the stomach in such a way that the final volume in each patient should reach 300 cc.

### Data collection from intervention group

After confirmation of the NG tube placement in the stomach, the lavage was performed, and the amount of gastric residual was measured and recorded, then this amount of lavaged food was returned to the stomach. In the next stage, abdominal massage was performed at 8 o’clock in the morning for 20 min, and after massage, the gavage was performed, and the gastric volume was increased to 300. After 2 h, the second stage of abdominal massage was performed, and finally, 1 h after the second massage, at 12 o’clock, the GRV was examined.

### Data collection from the control group

After confirmation of the insertion of NG tube into the stomach, lavage was performed for each patient at 8 am, and the GRV was measured and recorded, then this amount of lavaged food was returned to the stomach, and eventually, the volume of food in the stomach was increased to 300 cc by gavage, and 3 h later, at 12 o’clock, the GRV was checked and recorded.

### Ethical considerations

This study was registered in Iranian Registry of Clinical Trials, IRCT2017062134641N2. Also, the approval code from Ethics Committee of Kermanshah University of Medical Sciences was obtained, kums.rec.1396.31, and written informed consent was received from the individual accompanying the patient. The necessary assurance was given to the individual accompanying the patient and hospital officials about confidential information of patients and the anonymity right of them.

### Data analysis

Data were analyzed by SPSS version 24 and descriptive and inferential tests. Descriptive statistics such as frequency, frequency percentage, mean and median, and standard deviation were used for this purpose. The demographic information of the two groups was investigated based on qualitative variables (sex, marital status, educational level, and occupation) using chi-square test. Wilcoxon test was used to compare the mean of the intended quantitative feature before and after the intervention in each of the two control and test groups (to compare changes within groups). The U Mann-Whitney test was used to compare the mean rank of the quantitative intended feature in the two control and test groups before and after the intervention (in order to compare the variations between the groups). Independent and paired *t* tests were used to compare the mean of the total GRV in both groups before and after intervention. The significance level of the tests was considered 0.05.

## Results

In this study, 76 patients were recruited into the study, and among them, 16 (21%) were excluded because of death (3 patients), transfer to other hospital or ward (3 patients), NG tube extraction (3 patients), cardiopulmonary resuscitation (1 patient), discharge (2 patients), and change in feeding approach (4 patients). Thus, analysis was conducted onto 60 individuals (79%). Among the patients, 60% (36 patients) were male. The mean and standard deviation of age was 59.72 ± 16.02 years. The majority of the subjects in both groups were married (83.3%). Regarding the educational degree, most of the participants had diploma (30%), and the number of illiterate ones was less than the rest (8.3%). Regarding their occupation, most of them were unemployed (30%), and the number of those who were employed was less than the rest (8.3%). The intervention and control groups were similar in terms of demographic variables including gender, marital status, educational level, and employment status, and there was not any significant difference (*p* value < 0.05), and also, the two groups did not differ in terms of age (Table 1).

**Table 1:** Demographic characteristics of two groups based on the variables of sex, marital status, education, job, and age

Variables	Case *N* (%)	Control *N* (%)	Total *N* (%)	Statistical test
Sex				
Male	18 (60)	18 (60)	36 (60)	*χ*^2^=0 *p* = 0.604
Female	12 (40)	12 (40)	24 (40)	
Marital status				
Single	5 (16.7)	5 (16.7)	10 (16.7)	*χ*^2^=0 *p* = 0.635
Married	25 (83.3)	25 (83.3)	50 (83.3)	
Level of education				
Illiterate	3 (10)	2 (6.7)	5 (8.3)	*χ*^2^=1.132 *p* = 0.889
Elementary	8 (26.7)	6 (20)	14 (23.3)	
Diploma	8 (26.7)	10 (33)	18 (30)	
Associate	6 (20)	5 (16.7)	11 (18.3)	
Bachelor	5 (16.7)	7 (23)	12 (20)	
Employment status				
Unemployed	7 (23.3)	11 (36.7)	18 (30)	N/A
Retired	7 (23.3)	5 (16.7)	12 (20)	
Housewife	8 (26.7)	4 (13.3)	12 (20)	
Employee	2 (6.7)	3 (10)	5 (8.3)	
Free job	6 (20)	7 (23.3)	13 (21.7)	
Age (mean and SD)	60.76 ± 17.38	58.66 ± 14.75	59.72 ± 16.02	*t* = 0.508 *p* = 0.616

*N/A* not applicable

Comparing the mean of the total GRV before and after the intervention in both groups, the results showed that the mean of the total GRV before the intervention between the two groups was not statistically significant (*p* < 0.05). However, in comparison of the mean of the total GRV after the intervention in the case group (97.30 cc) was less than the control group (143.46 cc) (*p* value < 0.05, *t* = 3.62). In addition, the mean of GRV was not changed in case group before and after of intervention, significantly; however, it was increased in the control group (*p* < 0.001) (Table 2).

**Table 2:** Comparison of the total average of GRV before and after intervention in both groups

Groups Time of GRV measurement	Case group Mean (SD) of GRV (cc)	Control group Mean (SD) of GRV (cc)	Statistical test
Before	106.76 (58.56)	108.63 (26.58)	*t* = 0.159 *p* = 0.874
After	97.30 (54.06)	143.46 (39.93)	*t* = 3.62* p < 0.001
Statistical test	*t* = 0.964 *p* = 0.343	*t* = 4.70 *p* < 0.001*	

*is significant

Comparing the GRV before intervention in the case and control groups in different days, the results showed that the mean gastric volume before intervention in the case and control groups in different days had no significant difference (Table 3), but the mean of the GRV after the intervention in the case and control group was significant in the different days, and it was less in the case group in all 3 days (*p* value < 0.05) (Table 4).

**Table 3:** Comparison of the average of GRV before intervention in the case and control groups in different days

Variables	Groups	Average rating	Statistical test
Average of GRV before intervention in first day	Case	29.03	*p* = 0.514 *Z* = − 0.562
	Control	31.97	
Average of GRV before intervention in second day	Case	30.45	*p* = 0.982 *Z* = − 0.022
	Control	30.55	
Average of GRV before intervention in third day	Case	29.02	*p* = 0.509 *Z* = − 0.660
	Control	31.98

**Table 4:** Comparison of the average of GRV after intervention in the case and control groups in different days

Variables	Groups	Average rating	Statistical test
Average of GRV after intervention in first day	Case	21.52	*p < 0.001 *Z* = − 3/994
	Control	39.48	
Average of GRV after intervention in second day	Case	24.53	*p = 0.008 *Z* = − 2.651
	Control	36.47	
Average of GRV after intervention in third day	Case	20.25	*p < 0.001 *Z* = − 4.563
	Control	40.75	

*is significant

## Discussion

The results of this study showed that tensegrity type of abdominal massage can have an important effect on the reduction of GRV in patients hospitalized in intensive care units who are fed through NG tube, as the total mean of GRV in all days was significantly less than the control group, and also, it was low in all days in the case group. Controlling and reducing the GRV could be an important measure for improving nutritional status and reducing complications in critically ill patients [19], and consequently decreasing the rate of malnutrition [20]. Also, this measure could reduce the rate of vomiting and abdominal distention and improve weight gain and defecation pattern [17]. In this regard, a study by Kahraman and Ozdemir [16] in Turkey, conducted on the effect of abdominal massage on the GRV of patients hospitalized in intensive care unit, showed that the GRV on the last day compared to the first day had a significant reduction, but there was an increase in GRV in the control group. The results of this study are in line with our study. In a randomized control trial study by Dehghan et al. [21] in Iran on 70 patients by the tracheal tube, there were no differences between the two groups in terms of gastric residual volume after abdominal massage intervention. The results are not in line with our study, which may be related to the difference in massage time (15 min). In another similar study conducted by Uysal et al. [9] in İzmir on patients with neurology and neurosurgery, the results showed that in the test group, GRV was increased two times, and in the control group, it was more than eight times. The results of this study showed that the increase in the GRV in the control group was more than that in the case group, and the results were in line with our study. Another study was conducted by Warren [22] in America on the effect of abdominal massage on the GRV in patients connected to the ventilator system. The measurement results of the GRV in the first measurement, compared to the last one in the case group, showed a significant reduction in comparison to the control group, which is in line with the current research. In a study performed by Tekgündüz et al. [17] on premature infants, the results of the Wilcoxon test showed that the GRV in the case group, in the last day compared to the first day, had a significant decrease (*P* value< 0.05), but after comparing the experimental group with the control group in terms of the volume of residual stomach food, the results showed that there was no statistically significant difference. The results of other researches, such as the study by Uysal [23] on patients hospitalized in the neurosurgical department, represented the effect of abdominal massage on the gastric residual volume in patients hospitalized in intensive care units, which is in line with the present study.

Various studies shown that abdominal massage can play an important role in reducing the GRV, and it is through the stimulation mechanism of the peristaltic movements, intra-abdominal pressure changes, mechanical and reflexive effects on the intestines, shortening the food transition time in intestines, increased intestinal movements, and easier food flow through the digestive tract [9, 18]. Also, the effect of abdominal massage may be due to the parasympathetic stimulation that is followed by the stomach and intestine stimulation, and movements of digestive system increases, and this increase in activity leads to easier digestion of food in the stomach and its easier movement in the intestine. In a study on premature infants to show how massage leads to weight gain, the results of the study showed that abdominal massage increases the activity of vagus nerve and stomach movements [24]. In another study by McClurg et al. [25], which was conducted on a patient with multiple sclerosis, the results of the study showed that abdominal massage through the activation of parasympathetic divisions in the autonomic nervous system increased muscle movements in the intestine and increased digestive system secretions and relaxation of the sphincters of digestive tract, by affecting digestive system function, and had an important role in relieving constipation symptoms in these patients. Turan and Ast [26] also showed that abdominal massage by increasing peristalsis movements increases the intestinal movements, has a positive role in reducing constipation following surgery, and also increases the quality of life of postoperative patients.

Patients hospitalized in the intensive care unit connected to the ventilator, and also fed through the NG tube, due to aspiration of food following an increase in the GRV have pneumonia associated with ventilator. Regarding this, a study by Kahraman and Ozdemir [16] was conducted, and the results showed that pneumonia associated with ventilator in the group receiving abdominal massage was five times lower, although this result is not statistically significant compared to the control group, but abdominal massage can reduce the gastric residual volume and food regurgitation to esophagus, reduce the risk of aspirating this food into the lungs, and reduce the risk of pneumonia associated with mechanical ventilation in these patients [16]. Also, in another study by Le Blanc et al. [27] on patients undergoing colectomy with pain and ileus of the intestine, the results showed that the abdominal massage performed by a mechanical device also reduces the pain and also the duration of intestinal paralysis in these patients.

### Limitations

In this study, the researcher had a little knowledge about abdominal massage. Therefore, for this limitation, he was trained by a sports medicine specialist to perform abdominal massage. Also, it was not possible to obtain satisfaction of the patient due to their unconsciousness. Therefore, for this limitation, the consent of the individual accompanying the patient was sought, in case of referring to the hospital. In addition, we did not take any other information about some variables such as surgical/medical status, main diagnosis, organ failures, severity scores, elective/emergency admission, and requirement of mechanical ventilation and vasopressors, which may affect GRV in intensive care patients.

## Conclusion

The purpose of this study was to evaluate the effect of abdominal massage on the GRV in patients hospitalized in intensive care unit. The results of this study represented the effect of abdominal massage on reducing the gastric residual volume, so this procedure is recommended to be considered as a care method to improve nutrition status in patients hospitalized in these units.

## Abbreviations

GRV: Gastric residual volume
SD: Standard deviation
